# Status Quo in Mechanical Plaque Control Then and Now: A Review

**DOI:** 10.7759/cureus.28613

**Published:** 2022-08-31

**Authors:** Sumeet H Toshniwal, Amit Reche, Pavan Bajaj, Labdhi M Maloo

**Affiliations:** 1 Dentistry, Sharad Pawar Dental College & Hospital, Datta Meghe Institute of Medical Sciences (Deemed to be University), Wardha, IND; 2 Public Health Dentistry, Sharad Pawar Dental College & Hospital, Datta Meghe Institute of Medical Sciences (Deemed to be University), Wardha, IND; 3 Periodontics, Sharad Pawar Dental College & Hospital, Datta Meghe Institute of Medical Sciences (Deemed to be University), Wardha, IND

**Keywords:** interdental cleaning aids, power driven toothbrushes, plaque control, history of tooth brushes, smart toothbrushes, mechanical plaque control, toothbrushes

## Abstract

There are various kinds of dental diseases, of which dental caries and disease of periodontal origin are the most common. A very strong connection has been proven between oral inflammation and general health of an individual. Dental Biofilm is the main cause for gingivitis and periodontitis. Dental plaque may also be referred to as microbial plaque that consists of highly organized structures of different microbiotas attached to the hard tooth structure, which may be bound by salivary glycoproteins. Plaque control is a term that refers to removal of already formed or the control of formation of this microbial biofilm. There have been various methods practiced for plaque control; they are broadly classified into mechanical methods and chemical plaque control methods. Mechanical plaque control further includes many other methods such as manual toothbrushes and smart toothbrushes, which includes power-driven toothbrushes, sonic and ultrasonic toothbrushes, solar-powered toothbrushes (ionic toothbrushes), disposable toothbrushes, and laser toothbrushes; this also includes interdental cleaning aids. Continuous advancements with the integration of technology have been made in the field of mechanical plaque control to improve its quality.

## Introduction and background

There are various kinds of dental diseases, of which dental caries and disease of periodontal origin are the most common. According to the recent evidences, it is evident that there is a very strong connection between oral inflammation and general health of an individual [1]. Dental biofilm has been proven to be one of the main causative factors for gingivitis, which may further lead to periodontitis. Dental plaque also alters the ecology of the oral cavity by accumulation of microbes and may lead to advancement or formation of new carious lesions [2]. This dental plaque may also be referred to as microbial plaque that consists of highly organized structures of different microbiotas attached to the hard tooth structure, which may be bound by salivary glycoproteins. Plaque biofilm formation is a gradual process that includes following steps: (1) formation of pellicle, (2) primary colonization, (3) secondary colonization, and (4) biofilm maturation [3]. In order to control the formation of this microbial biofilm, it is necessary to practise oral hygiene measures on a day-to-day basis [2]. Plaque control is a term that refers to removal of already formed or the control of formation of this microbial biofilm [3].

## Review

There have been various methods practiced for plaque control, which may be broadly classified into mechanical methods and chemical plaque control methods.

Mechanical Plaque Control

Mechanical disorganization or removal of the plaque has been proved to be the most commonly used method for plaque control, in which using toothbrushes has the main share [4].

Classification of mechanical plaque control aids

Classification of mechanical plague control aids is been tabulated in Table 1.

**Table 1:** Classification of mechanical plaque control aids

Chewing twigs	Toothbrushes	Interdental aids
Neem twigs	Manual toothbrushes	Interdental floss
Mango twigs	Power-driven toothbrushes	Interdental brushes
Miswak twigs	Sonic and ultrasonic toothbrushes	Wooden tips
		Rubber tips

History of toothbrushes

Earlier, twigs were used in place of toothbrushes. In India, before toothbrushes were invented, a twig of Neem plant, which is said to be having medicinal properties, was widely used for toothbrushing. An Aromatic twig of plant called Miswak was also in wide use as a toothbrush in Middle eastern region. A twig of plant from the Salvadoraceae family of plants was also used commonly as an alternative to Neem; it was chewed until its ends frayed [5-6]. Historians trace the evidence of the first toothbrush to be developed by China in 1498 C.E. The bristle brushes were reinvented lately in the 18th and early 19th centuries. Lately, in 1930s, nylon filaments, which were less expensive, replaced natural bristles, and plastic and wood replaced the bone handles; later in the year 1960, soft nylon bristle brushes came into existence [5-6]. Electric toothbrushes were first introduced in the early 1960s. They were introduced to improve outcomes of poor teeth brushing practice. Smart toothbrushes have recently flourished due to their decreased cost and increased efficiency.

Manual toothbrushes

These are the most commonly used toothbrushes. In 1980s, a single type of manual brush was marketed, which had a multitufted toothbrush head shape. Then, in 1990s, a varied type of toothbrushes was invented, which had different types of head designs, angulations, shapes, sizes, and different types of bristles. These modifications made the plaque removal by these toothbrushes more easier and more efficient. There have been many modifications in the design of manual toothbrushes, such as handle of the toothbrushes, bristles of toothbrushes, and shape and size of toothbrush heads. Currently, a variety of toothbrushes are available, which may have difference in their design, for example, they may be compact or normal and their bristles may be soft, medium, or hard [1].

According to the American Dental Association (ADA), a toothbrush should have a set of specifications, which are as follows: 1) length of the toothbrush should be 1-1.25 inches, (2) width of the toothbrush should be 5/16 to 3/8 inches, (3) surface area of the toothbrush head should be 2.54 to 3.2 cm, (4) there should be two to four rows of bristles, (5) it should have 5-12 tufts per row, and (6) there should be 80-85 bristles per tuft [4-7].

Conventional Flat Trim Toothbrushes

This is the gold standard and the most commonly used toothbrush to maintain oral hygiene. This toothbrush complies with the ADA specifications and contains bristles placed at the same level, which may compromise tooth cleaning and plague removal due to difference in tooth placement in every individual [4-6]. Modifications are made in the bristle designs and mechanism to overcome the drawbacks of this toothbrush, which are elaborated further.

Smart toothbrushes

Toothbrushes since the time they were invented are continuously been modified to increase their efficiency [5]. Continuous research and development have led to development and modifications of toothbrushes such as power-driven toothbrushes, rotating and oscillating toothbrushes, sonic and ultrasonic toothbrushes, solar-powered toothbrushes, and disposable toothbrushes [2].

Power-Driven Tooth brushes

Power-driven toothbrushes were first invented in 1960s, which were the first alternative to manual toothbrushes [2].These electric toothbrushes have been found to be efficient in removing plaque as compared to other manual toothbrushes in various studies carried out across the globe. They were proved to be useful for the handicapped and for people with some or the other mental dexterity. Studies also claimed these to be efficient in removal of the plaque in children [4]. These power-driven toothbrushes normally have a compact head as compared to manual toothbrushes and have bristles arranged in a circular pattern [2]. Power toothbrushes with timer are also available, which lets an individual spend a proper time on brushing, which is very necessary in order to maintain oral health. Greater efficiency, reduced manual work, and reduced cost have led to increasing use of these rotating oscillation toothbrushes [7,8].

Sonic and Ultrasonic Toothbrushes

Ultrasonic toothbrushes are manual toothbrushes with embedded pizoelectric emitter in the brush head. Emmi®-dent was the first ultrasonic toothbrush that used nano paste technology [2]. The core technology used in ultrasonic toothbrushes is the ultrasonic vibration of the bristles [3]. These ultrasonic toothbrushes help in reducing plaque and periodontal disease by destroying the bacteria; they are also helpful in removing the stains of coffee, wine, nicotine, and food [8]. There is not much difference noticed in the sonic and ultrasonic toothbrushes with respect to plaque removal, and both of them showed significant results [3]. These ultrasonic toothbrushes can be useful for patients undergoing orthodontic treatment and after periodontal surgery [8,9].

Solar-Powered Toothbrushes (Ionic Toothbrushes)

Solar-powered toothbrush was first invented in Japan by the name of Soladey-3. It has a solar panel that is attached to the toothbrush handle, which absorbs electrons from light and transmits them to the teeth, which, in turn, reacts with saliva to remove the plaque attached to the teeth. It is totally different from powered toothbrushes, which use toothpaste to remove the plaque [8].

Disposable Toothbrushes

These are toothbrushes that are meant only for one-time use and have disposable heads that prove to be very useful for travellers, children, and hospital patients [2].

Chewable toothbrushes: These are very small plastic-molded toothbrushes that are used when no water is available. These toothbrushes should not be swallowed. They are available in different flavors and are commonly available in bathroom-vending machines [8].

Laser Toothbrushes

Dentinal hypersensitivity has been one of the most prevalent problems. Laser stops the conduction of impulse through dentinal tubules and thus helps in reducing dentinal hypersensitivity. Laser toothbrushes are a modified version of power-driven toothbrushes, which emits laser and thus helps reduce hypersensitivity, and it can be done at home [2].

Biodegradable Toothbrush

“Bogobrush” is a biodegradable toothbrush made up of wood commonly known as “bamboo toothbrush.” It is a type of biodegradable toothbrush that can be disposed of without harming the nature as compared to toothbrushes made up of plastic. The main aim of these toothbrushes is to make it nature-friendly. It is been marketed in different types such as foldable toothbrushes and ones with plant seeds within the toothbrush handle that when buried grows into a plant [10].

Polyhydroxyalkanoate (PHA)-based biodegradable manual toothbrush is another type of toothbrush that has a hollow handle and is biodegradable in nature [11].

Curved Bristle Toothbrushes

These are specific toothbrushes with curved bristles and are proven to be highly effective in the maintenance of oral hygiene in handicapped people. This curved bristle toothbrush consists soft curved bristles, which occupy the occlusal and proximal surfaces of tooth covering the complete tooth. This toothbrush requires only horizontal motion rather than vertical motion, which is proved helpful for handicapped people; another advantage of these toothbrushes is that they do not have sharp bristle ends, which prevent harming gingiva [12].

Specialized Orthodontic Toothbrushes

People undergoing fixed orthodontic treatment have braces, bands, and wires placed on their tooth, which may make it difficult for them to brush properly and maintain proper oral hygiene by using conventional toothbrushes, Thus, orthodontic toothbrushes are designed differently from conventional toothbrushes. The length of bristles is shorter in orthobrushes, bristles are arranged in a V shape and in a zigzag pattern, and they are inclined to facilitate proper cleaning. Many studies have shown that oral hygiene maintenance was improved in orthodontic patients after using specialized orthodontic brushes [13,14].

Toothbrush With Modified Bristle Designs

Toothbrushes are constantly modified to improve their cleaning efficacy, and modifications are made to their bristle designs and head angulations [15]. Toothbrushes with different bristle designs are currently commercially available, such as toothbrushes with flat trimmed bristles, those with wavy bristles, those with beveled bristles, and those with zigzag bristles.

Toothbrushes With Infused Minerals

Recently, many toothbrushes are available commercially with infused minerals in them, which may be useful in maintaining oral hygiene and improving gingival health. These toothbrushes include charcoal-infused toothbrushes, Neem-infused toothbrushes, and so on [16].

**Figure 1:**
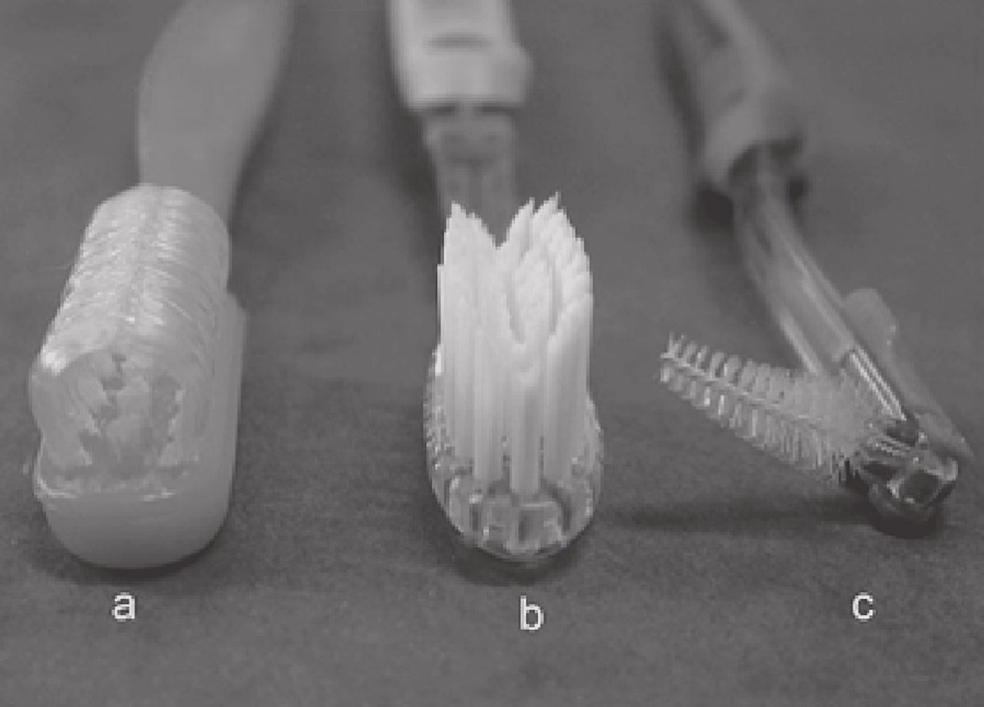
(a) Curved bristle toothbrush. (b) Orthodontic toothbrush. (c) Interproximal bottle brush. [17]

Toothbrushes with different bristle designs are depicted in Figure 1 [17].

Interdental cleaning aids

Most of the periodontal diseases originate interdentally, as toothbrushing is not adequate for interdental cleaning and thus it is necessary to use interdental cleaning aids [1]. Toothbrushing is one of the main methods and the most commonly used methods to control plaque, but eminence of periodontal diseases stays the same [18]. Toothbrushing alone might not be sufficient in order to control the spread of periodontal diseases, as there may be seen stagnation of bacteria in the interproximal areas of all the teeth. Toothbrushes are mainly effective in disengaging plaque and adherent bacteria from the flatter surfaces of tooth such as buccal and lingual surfaces. Interdental cleaning aids are useful in order to clean the proximal interdental areas [19,20].

Interdental Brushes

Interdental brushes were developed as an alternative to the wooden sticks for interdental cleaning in the 1960s [2]. These interdental brushes have diameters slightly more than the gingival embrasure, exert pressure on surfaces of teeth in wide interproximal space, and help in achieving better plaque control and removal than sticks or floss [1]. Interdental brushes are made of nylon bristles which, were twisted across as a wire, but metals were uncomfortable and thus currently these are twisted across plastic points. These brushes are available in different types such as conical or cylindrical and in different sizes that are color-coded for convenience [2].

Dental Floss

Dental floss is one of the most widely used methods of interdental cleaning. It is used as an adjunct to toothbrushing. Different types of dental floss have been marketed currently, which include floss with and without handle, flavored and unflavored floss, waxed and unwaxed floss, and floss classified according to the diameter such as thin, medium, and thick. Recently, with the integration of technology, powered flossing devices have also come into use.

Powered flossing devices: In order to overcome the drawbacks of manual flossing, power flossing devices were invented, as manual flossing required knowledge and proper technique. These powered flossing devices included battery-operated nylon tip which slips interdentally [2].

Single Tufted Brushes

These are toothbrushes with a single tuft and are primarily used to clean the tooth along the gum line. These are most commonly used in patient with prosthesis or patient undergoing orthodontic treatment. They may be straight or contra angle type [2].

Oral Irrigators

**Figure 2:**
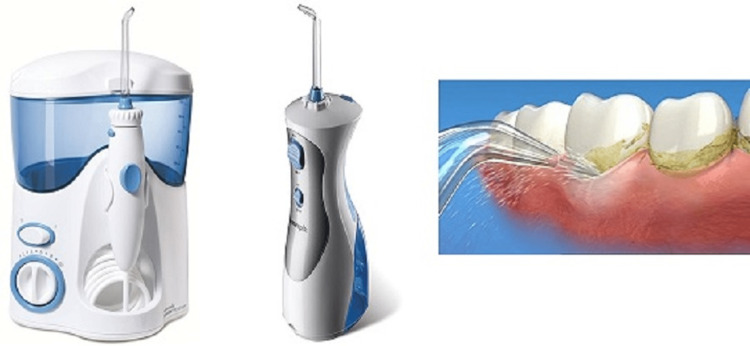
Oral irrigators

These are irrigation devices that deliver a pulsating stream of fluid along the interdental area and may also be used to deliver medication such as chlorhexidine, antibiotics, and 5% tetracycline. These oral irrigation devices are of different types, which may be power- and nonpower-driven, designed for home or professional care, and may be with or without replaceable tips [2]. Refer to Figure 2 for oral irrigators.

## Conclusions

Maintaining oral hygiene is most important in order to stop the progression of many diseases. Plaque control is described to be one of the most integral methods to control the progression of bacteria. Mechanical plaque control is the primary method to remove and control plaque, where toothbrushes have a prime role. We have evolved a lot from sticks to brushes, and now integrating technology in this field will prove to be promising in improving the overall oral health of an individual.

## References

[1] Harrison P (2017). Plaque control and oral hygiene methods. J Ir Dent Assoc.

[2] Mandal A, Singh DK, Siddiqui H, Das D, Dey AK (2017). New dimensions in mechanical plaque control: an overview. Indian J Dent Sci.

[3] Digel I, Kern I, Geenen EM, Akimbekov N (2020). Dental plaque removal by ultrasonic toothbrushes. Dent J (Basel).

[4] Choo A, Delac DM, Messer LB (2001). Oral hygiene measures and promotion: review and considerations. Aust Dent J.

[5] Fischman SL (1997). The history of oral hygiene products: how far have we come in 6000 years?. Periodontol 2000.

[6] Tadinada A, Kilham J, Bysani P (2015). The evolution of a tooth brush: from antiquity to present- a mini-review. J Dent Health Oral Disord Ther.

[7] Menon L, Ramamurthy J. (2014). New vistas in plaque control. IOSR J Dent Med Sci.

[8] Sharma K, Sangwan A. (2013). Era of smart toothbrushes. Adv Hum Biol.

[9] Iacono VJ, Aldredge WA, Lucks H, Schwartzstein S (1998). Modern supragingival plaque control. Int Dent J.

[10] Kmetz B, Magyar B, Szabó FJ. (2018). Biodegradable Toothbrush. Des Mach Struct.

[11] Das A, Goswamy M, Mahajani M, Choukse V, Patil KS, Chavhan SS (2022). Various latest toothbrush design. Int J Med Oral Res.

[12] Chava VK (2000). An evaluation of the efficacy of a curved bristle and conventional toothbrush. A comparative clinical study. J Periodontol.

[13] (2022). Nicolas YM.. https://patentimages.storage.googleapis.com/dc/16/9e/4dedfe694023eb/US4706322.pdf.

[14] Muhammad N, Abdullah K, Faiza S, Farzeen T, Navaid Q, Rashid AS, Mutaal M (2015). The efficacy of orthodontic brushes, their effect on the duration of orthodontic treatment and on the periodontal health among orthodontic patients. Int J Multidiscip Health Sci.

[15] Sripriya N, Shaik Hyder Ali KH (2007). A comparative study of the efficacy of four different bristle designs of tooth brushes in plaque removal. J Indian Soc Pedod Prev Dent.

[16] AlDhawi RZ, AlNaqa NH, Tashkandi OE, Gamal AT, AlShammery HF, Eltom SM (2020). Antimicrobial efficacy of charcoal vs. non-charcoal toothbrushes: a randomized controlled study. J Int Soc Prev Community Dent.

[17] Arici S, Alkan A, Arici N (2007). Comparison of different toothbrushing protocols in poor-toothbrushing orthodontic patients. Eur J Orthod.

[18] Ng E, Lim LP (2019). An overview of different interdental cleaning aids and their effectiveness. Dent J (Basel).

[19] Claydon NC (2008). Current concepts in toothbrushing and interdental cleaning. Periodontol 2000.

[20] Tarannum F, Faizuddin M, Swamy S, Hemalata M (2012). Efficacy of a new interdental cleaning aid. J Indian Soc Periodontol.

